# Does early intervention improve outcomes in physiotherapy management of lumbar radicular syndrome? A mixed-methods study protocol

**DOI:** 10.1136/bmjopen-2016-014422

**Published:** 2017-03-03

**Authors:** Michael Reddington, Stephen J Walters, Judith Cohen, Susan Baxter

**Affiliations:** 1 Therapy Services Outpatient Department, Northern General Hospital, Sheffield, UK; 2 Designs, Trials and Statistics ScHARR, University of Sheffield, Sheffield, UK; 3 Clinical Trials Research Unit ScHARR, University of Sheffield, Sheffield, UK; 4 Section of Public Health ScHARR, University of Sheffield, Sheffield, UK

**Keywords:** QUALITATIVE RESEARCH

## Abstract

**Introduction:**

Lumbar radicular syndrome (LRS) can be a painful and debilitating condition. The optimum management strategies and their timing remain elusive despite extensive research. Surgery provides good short-term outcomes but has concomitant risks and costs. Physiotherapy is commonly practised for patients with LRS but its effects remain equivocal and there is a lack of consensus on the type, duration and timing of physiotherapy intervention. There is a lack of high-quality evidence into new and innovative management strategies and the timings of those strategies for LRS. This pilot trial is an essential preliminary to a definitive randomised controlled trial (RCT) assessing the effectiveness and cost-effectiveness of early physiotherapy intervention for patients with LRS. The study will test the protocol, the intervention, the use of outcome measures and the ability to set-up and run the trial to enable refinement of a future definitive RCT.

**Methods and analysis:**

This is a mixed-methods study encompassing an external pilot RCT with integrated qualitative interviews with patients, clinicians and other key stakeholders. 80 patients will be recruited from primary care and randomised, after consent into 1 of 2 groups. Both groups will receive individually tailored, goal orientated physiotherapy. The usual care group will begin their physiotherapy 6 weeks after randomisation and the intervention group at 2 weeks after randomisation. Outcome measures will primarily be feasibility parameters including the ability to recruit and retain patients and to deliver the intervention. Data will be collected at baseline, and 6, 12 and 26 weeks following randomisation.

**Ethics and dissemination:**

The study has received favourable ethical review from the East of Scotland Research Ethics Service (EoSRES) on the 20 August 2015 (15/ES/0130). Recruitment began on the 1 March 2016 and is expected to close in January 2017. Data collection is anticipated to be complete in July 2017. The study results will be made available to participants, clinicians involved in the study and the wider clinical community through publication in a peer reviewed journal and at conference presentations.

**Trial registration number:**

ISRCTN: 25018352, Pre-results; Clinical Trials.Gov: NCT02618278 Document version V1.1 23.9.2016.

**Strengths and limitations of this study**

- This feasibility study will inform the design and conduct of a definitive randomised controlled trial of an early physiotherapy intervention for the treatment of lumbar radicular syndrome (LRS).
- The intervention is a safer, cheaper alternative to surgery for patients with LRS and should be easy to adopt and deliver within current treatment pathways.
- The qualitative component of the study will enable patient and stakeholder views to be taken into consideration and they will be used to inform modifications to the intervention and design of the definitive trial.
- Recruitment in the feasibility study will be from 14 General Practitioner (G.P) practices in the north of England with diverse demographic populations. Generalisability of the results will need to be considered when planning a larger trial including other areas of the UK.

## Introduction

Lumbar radicular syndrome (LRS) is a painful and often disabling condition, which is usually of benign causation. It can be self-limiting and may last a short time with no significant sequelae or, in some individuals it can be severely painful and disabling in the long term. The reasons for the wide variations in presentation, outcome and duration in patients are not fully understood.^1^

LRS is perhaps better known as sciatica, although sciatica does not encompass neural dysfunction from the upper lumbar nerve roots and thus, the term LRS is preferred. LRS is defined as leg pain in an area served by one or more spinal nerve roots and can be accompanied by neurological deficit such as paraesthesia, anaesthesia and myotomal weakness.^2^ There are many and varied estimates of prevalence of LRS ranging from 1% to 43%.^3^

LRS is a major cause of disability, work loss and presentation to healthcare.^4^ The Health Council of the Netherlands estimated a cost of 2.9 billion Guilders to the Dutch economy in 1999 (around €1.5 billion at September 2015 exchange rate). The costs of surgery for LRS (microdiscectomy and laminectomy) were found to add $5 billion to the overall cost of back pain and LRS in the USA in 2004.^5^ The cost of physiotherapy for a group of patients who went on to have surgery for LRS accounted for 11% of the total preoperative costs or on average $379 per patient. The mean cost of imaging alone accounted for 31% of costs or $1067 for each patient,^6^ illustrating the relatively small costs of physiotherapy compared with other, non-therapeutic costs.

The management of LRS is a much debated and contentious area. Surgery for patients with LRS has been advocated, with good reported outcome.^7^ However, significant number of patients never have any substantial relief from surgery,^8^ ^9^ with unsatisfactory outcomes in over 20% of patients at 5 years, irrespective of the type of treatment they receive, including surgery.^10^ Although, it is not known with any certainty how long to wait to allow spontaneous resorption of the inter vertebral disc (IVD) prolapse, it has been suggested that by 12 weeks 75% of LRS sufferers will have symptomatically resolved.^11^ The favourable effects of conservative treatment for LRS have been demonstrated with 90% of patients improving within 12 weeks after onset.^12^ A ‘wait and see’ approach is often advocated but a significant number of people suffering with LRS do not recover in the short or medium term and the optimal window for surgery can then be missed. Lewis *et al*^13^ note the importance of early treatment in order to prevent chronic symptom development and the ensuing resistance to treatment and costs.

While physiotherapy for LRS has been advocated, there is a lack of consensus on the type, duration or timing of intervention.^14^ There are numerous physiotherapy approaches to low back pain (LBP) and LRS, none of which have significant degree of scientific rigour surrounding them.

It is known that patients prefer and have improved outcomes with early intervention physiotherapy for LBP.^15–17^ Delayed initiation of physiotherapy for patients with LBP in primary care is associated with increased cost and increased healthcare consumption.^18^ These findings were echoed by Gellhorn^19^ who found that patients who received physiotherapy <4 weeks after onset of their LBP had lower healthcare usage (and associated costs) than those who received physiotherapy more than 3 months after onset. Although such evidence does not directly exist for the provision of physiotherapy for patients with LRS, it may be suggested that early intervention is preferable.

### Aims and objectives

The overall aim for the study is to investigate the feasibility of undertaking a fully powered, multicentre randomised controlled trial (RCT) to determine the effectiveness and cost-effectiveness of early intervention physiotherapy for patients with LRS.

### Objectives

The objectives of the pilot trial fall into two categories. First, process objectives will allow the analysis of the practical and logistical aspects of setting up and running the study. Second, the feasibility objectives will provide data on recruitment, the use of outcome measures, randomisation and data collection and the delivery of the training and intervention. This information will be used to inform the definitive RCT.

### Process objectives

1. To test the feasibility, practicality, safety and acceptability of the study design and protocol.
2. Demonstrate the ability to set-up and recruit in primary care centres.
3. To assess the feasibility of delivering the early intervention within the time parameters (2 weeks for the intervention group, 6 weeks for the usual care group).
4. Demonstrate a recruitment rate of seven patients per month in a maximum of 14 G.P centres, equal to a rate of 0.5 of a participant per centre, per month.
5. Demonstrate the ability to organise 75% of physiotherapy appointments within 2 weeks of randomisation.
6. Patient attendance at 66% of individual sessions.
7. 75% of patients randomised to early intervention have their first session within 20 days of randomisation.
8. Patient attrition rate of <25% over the course of the study.
9. Outcome measurement return rate of 80% at 6/52 follow-up.

### Feasibility objectives

1. To determine the acceptability of the intervention to patients and clinicians.
2. Demonstrate acceptability of the primary and secondary outcome measures to patients and clinicians.
3. To inform the sample size calculation for the definitive trial.

## Methods

### Design

Mixed-methods study comprising an external pilot RCT augmented by semistructured interviews with key stakeholders.

### Quantitative

Recruitment will occur in three, 20-week cycles, illustrated in table 1. In each of the three cycles there will be 12 weeks of active recruitment followed by up to 8 weeks for treatment to be completed. A 2-week period between cycles will provide time to reflect and analyse on the results from the stakeholder interviews and other feedback.

**Table 1:** Recruitment cycles

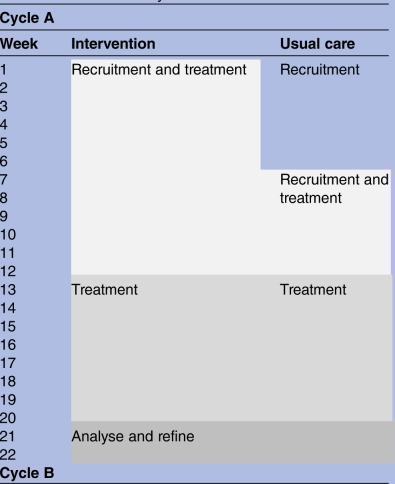

### Qualitative

In-depth, semistructured interviews will be carried out with consenting patients in the intervention and control arms of the study. This will involve interviewing the patients before and after the intervention delivery. The treating physiotherapists will be interviewed before they begin 3 days of intervention training and also at the completion of the study. Other stakeholders in the process of delivering the intervention will also be asked for their feedback during the course of the study either through interviews or in the case of G.Ps, a weekly email forum. The aims of the qualitative elements of the study are first to compare and contrast the experiences of patients in the intervention and control groups of the pilot trial, and second to obtain the views and experiences of patients with regards to the study processes. In particular, the design, methods, randomisation and intervention.

**Figure 1:**
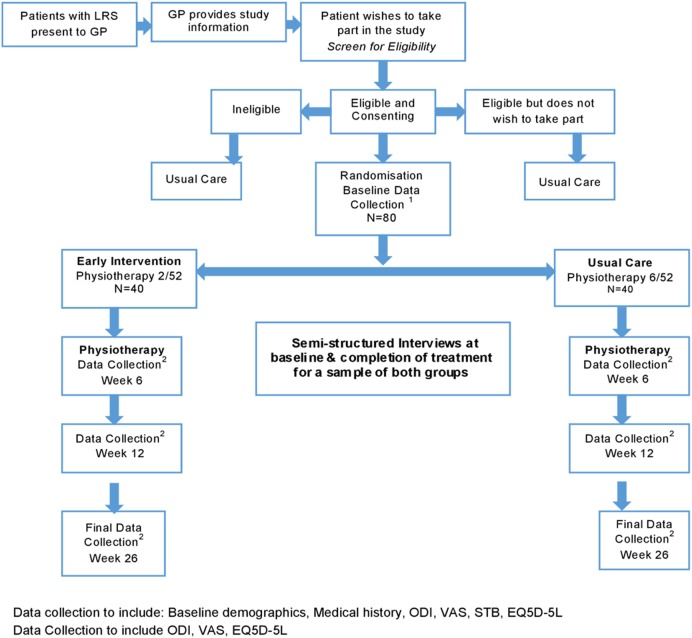
Flow chart illustrating the recruitment and randomisation process. GP, general practice; LRS, lumbar radicular syndrome; ODI, Oswestry Disability Index; STB, STarT Back screening tool.

A total of 80 patients with LRS will be recruited and each individual will be involved in the study for 6 months. A flow chart illustrating the study is found in figure 1.

### Ethical review and trial registration

The study is being conducted in accordance with the Declaration of Helsinki and local governance requirements. The trial has been registered with current controlled trials ISCTRN number: 25018352 and Clinical Trials.Gov number NCT02618278.

### Setting

Recruitment into the study will take place in a primary care setting with 14 G.P practices having committed to act as recruitment centres. The physiotherapy intervention will take place at one existing primary care treatment site.

### Participants (inclusion and exclusion criteria)

Patients will be eligible to participate in the study if they are over 18 years of age and have unilateral LRS. Patients will be ineligible to participate if they have any existing condition which will affect their ability to undergo rehabilitation, for instance previous Cerebrovascular accident (CVA) with physical and/or psychological sequelae which would prevent rehabilitation, proven vascular claudication of lower limbs which could mimic LRS, spinal fracture, ongoing cancer and inadequate English language skills preventing questionnaire and interview completion.

### Sample size

It has been recommend that an external pilot study has at least 70 measured subjects (35 per group) when estimating the SD for a continuous outcome.^20^ A sample size of 80 patients, with ∼10% allowance for lost to follow-up allows the SD of an outcome to be estimated to within a precision of ∼±16% of its true underlying value with 95% CI.

### Qualitative sampling

Although it is difficult to judge how many participants will be required for interview until data saturation is reached, it is estimated that around 10–15 interviewees will be required per study arm. Ritchie^21^ outlines several factors that can influence decision making regarding the size of samples in qualitative research. Two key factors are the heterogeneity of the study population and the available resources. It is expected that in this study population there should be a degree of homogeneity, in that all of the participants are suffering with LRS. Individual characteristics such as sex, age and duration of onset will be considered in order to achieve diversity in the sample.

### Recruitment and consent

Potential participants will be given a patient information sheet when they see their G.P for help with their LRS. A brief screening process will take place when the potential participant contacts the research team and consent will obtained during a face-to-face meeting, before baseline measures are collected.

### Randomisation

Information from the baseline data set will be used to randomise the participant. Randomisation will be achieved by a web-based randomisation system with one stratification factor (Oswestry Disability Index (ODI)) with three levels based on ODI severity; ‘mild & moderate’ (≤22–40%), ‘severe’ (>40–60%) and ‘crippled’ (>60–80%). A blinded block size will be used to minimise predictability. The participants will be informed, within one working day of their consent and randomisation of their group allocation. With a study of a complex intervention such as the one described, it is very difficult to blind either patients or clinicians to the treatment allocation, as it is obvious to clinician and patient that they are receiving an intervention at either 2 weeks or 6 weeks. In an effort to minimise bias, both groups of patients will receive treatment based on the same assessment and treatment framework.

### The intervention

The goal-orientated physiotherapy regimen for both groups will be tailored to the individuals' requirements. Participants will be assessed using a biopsychosocial approach based on seven different elements; neurological dysfunction,^22^ motor control of movement of the lumbar spine and pelvis,^23^ ^24^ movement restriction in the lumbar spine and pelvis,^25^ psychological barriers to recovery^26–28^ advice and education^29^ ^30^ functional-based exercise^31^ and pain.^32^ Participants will receive up to six sessions of physiotherapy over an 8-week period or until they have achieved their predetermined goals.

The seven elements of assessment and treatment are neither exclusive nor exhaustive but reflect the complexity of the clinical reality of a patient presenting with LRS. The Medical Research Council (MRC) guidance for developing and evaluating complex interventions has, therefore, been adopted as the principal framework around which the study has been based.^33^

Implementation fidelity testing will be carried out in order to assess the treating clinicians are delivering what is intended by the protocol.^34^ An independent assessor will review video footage of physiotherapist and participant session in order to assess implementation fidelity.

Patients are allowed to withdraw from treatment at any point and this will have no detrimental effects on their ongoing healthcare.

### Physiotherapist training and support

Three physiotherapists will be recruited from the physiotherapy service provider. They will undergo 3-day (20 hours) training in each of the seven elements of the assessment and intervention to promote and facilitate self-management, function, pacing advice and optimum analgesic advice together with equipping the patient to cope with their symptoms optimally.

### Data collection

Patients will be asked to complete baseline self-report and screening measures outlined in table 2 at the time of consent by the chief investigator or research nurse. These will include an initial self-assessment form including anthropometric data including gender, height, weight, socioeconomic status (work and any time off work due to the LRS). Participants will also be asked about their specific functional goals of undertaking physiotherapy. This baseline data will be used, in conjunction with a clinical assessment undertaken at their initial assessment, to formulate an individual treatment plan.

**Table 2:** Screening and outcome measurement collection

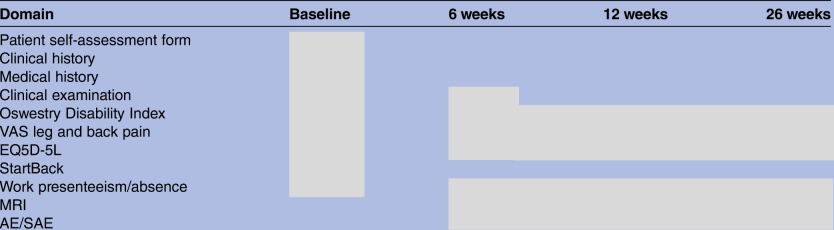

AE, adverse events; EQ5D-5L, EuroQol 5 dimension 5 level; SAE, serious adverse events; VAS, visual analogue score.

### Data management

Patient identifiable data will be entered onto a bespoke, secure study database provided by the University of Sheffield (UoS) Clinical Trials Research Unit (CTRU), who adheres to Standard Operating Procedures (SOPs) relating to all aspects of data management including data protection and archiving. Patients will be given a unique study number on consenting to enter the study and they will be identified using their participant study number only. All hard copy data will be stored in a locked filing cabinet in accordance with data protection requirements for the retention of research data and UoS and Sheffield Teaching Hospitals NHS Foundation Trust (STHFT) data management policies. Data will not be shared with anyone outside of the direct research team (chief investigator, CTRU and supervisors). Access to data, including the trial master file, will be restricted to the sponsor.

### Primary and secondary outcome measures

The primary outcome measures for the study will evaluate the feasibility of a range of factors. These will include the recruitment of patients into the study in a timely manner, in particular the ability to identify eligible patients and their willingness to be randomised. The recruitment rate and initiation of treatment within 2 weeks for intervention arm and 6 weeks for usual care arm will be noted. Ensuring patient safety and acceptability of the intervention to patients and clinicians and acceptability of the trial protocol. The practicalities of providing the physiotherapy and patient adherence to the intervention and in addition, the number of patients being referred into the secondary care system will be noted and costed accordingly. The number of patients undergoing surgery for their LRS will be recorded and costed as will the number of adverse events (AE) and serious adverse events (SAE). Finally, the time taken from randomisation to physiotherapy treatment initiation will also be recorded.

### Data analysis plan

As the trial is a pragmatic parallel group RCT, data will be reported and presented according to the CONSORT statement.^35^ The statistical analyses will be performed on an intention-to-treat basis. As a feasibility study, the main analysis will be mainly descriptive and will focus on CI estimation and not formal hypothesis testing. We will report rates of consent, recruitment and follow-up by centre and by randomised group. Outcome measures will be summarised overall and by randomised group. We will use the data from this feasibility study to estimate the consent rate, attrition rate, and the variability of the continuous outcomes (eg, leg and back pain VAS, ODI, EQ-5D) in the trial population and use this information to inform the sample size calculation for the definitive RCT. Since the intervention is therapist led we shall also use the data to estimate the intra-cluster correlation (ICC). We will also include, as part of the feasibility analysis, estimation of the effect size for the 12-week VAS-pain outcome (the probable primary end point for the definitive study) with CI estimates to check that the likely effect is within a clinically relevant range (as confirmation that it is worth progressing with the full trial). This information along with the acceptability of the study design and protocol to patients, therapists and G.Ps; the safety of the intervention; patient recruitment and consent/retention rates will enable us to determine whether or not the definitive RCT is feasible, within a satisfactory timescale and cost envelope using the UK centres alone. The time from randomisation to start of physiotherapy will be summarised by randomised group.

### Qualitative analysis

The constant comparative method will be used for the analysis of the interview data.^36^ A preliminary thematic analysis^37^ will be undertaken after three interviews by the lead author. This will take the form of category generation by defining units of data code generated by the interviews. Category generation will draw on both the interview data from the interviewee as well as the notes and reflections of the interviewer. The former aims to gain an insight of the meanings, views and opinions of the interviewee. The latter allows to allow the researcher to build on insights drawn from the interview in terms of the research area being addressed.^38^

Findings from interviews and feedback from participants and clinicians will be used after the 1st cycle of recruitment to inform changes to the intervention and processes for the 2nd cycle of recruitment. This will be repeated for the 2nd recruitment cycle to inform the 3rd cycle of recruitment. In this way it is envisaged that at the end of the 3rd and final cycle, the processes, intervention and design will be ready to form the basis of a full RCT. Changes will be made in discussion with the trial management group (TMG) and protocol amendments requiring ethical approval will be sought as appropriate. Subsequent interviews will be analysed by the researcher on an iterative basis, whereby, the ongoing analysis, along with the reflexive diary, will be used to identify new issues to explore with successive interviewees. Atlas.ti software will be used to manage the qualitative data.

### Mixed methods

A mixed-methods approach has been adopted in order to gain an insight into the defined feasibility objectives and to add depth of understanding as to why aspects of the intervention or study process are working or not. In doing so it is hoped that more than a sum of the parts of the quantitative and qualitative components of the research alone will be realised.

Integration of the findings of the quantitative and qualitative components, gathered throughout the study will occur at the analysis stage. A mixed-methods matrix^39^ will be used for analysis of data from individual cases in terms of quantitative and qualitative findings.

### Health economic analysis

An exploratory health economic analysis will be undertaken to inform a full-economic analysis for the future, full-scale trial. Patients will be asked to complete a short, self-report survey on costs that they have incurred as a direct consequence of their LRS. The factors on the analysis include time absent from work, analgesic requirements and cost, cost of any adjuncts used and costs incurred as a result of paying for care.

### Trial organisation and management

The study is sponsored by STHFT in collaboration with the School of Health and Related Research (ScHARR), the UoS. The lead researcher (MR) will act as chief investigator with responsibility for project management in accordance with STHFT and UoS SOPs and will be overseen by the TMG.

A Trial Steering Committee (TSC) will be appointed and comprise of an independent chair, two patient representatives, a general practice (GP) representative, and clinicians with experience and expertise in the field of spinal disorders, including LRS. The TSC will review safety and progress of the trial and make recommendations to the TMG about study continuation.

A Data Monitoring and Ethics Committee (DMEC) is not required as physiotherapy is routine treatment for this patient group, the intervention changes the timing only and as such was considered low risk.

### Dissemination

The results of the study will be presented initially to the patients who have taken part in the study and the participating clinicians. A report will be submitted to the funder of the study outlining the process and results. The results will be available via the study website at the host institute: http://www.sheffield.ac.uk/scharr/sections/dts/ctru/polar. The results will be presented nationally/ internationally at conferences and through peer review, open access journals, subject to acceptance. Furthermore, the findings will be presented locally at musculoskeletal training events for physiotherapists and G.Ps. A key facts summary will be provided for the local musculoskeletal service commissioners.

## Discussion

There is a lack of high-quality evidence about the optimal way to treat patients with LRS. This study is a preliminary step towards establishing if a novel physiotherapy intervention, delivered within 2 weeks of referral from the G.P is an effective treatment for these patients. This study has been designed to assess the feasibility of a future, full-scale trial and will not investigate effectiveness of the intervention. The definitive RCT will be informed by the results of the study and will investigate the effectiveness and cost-effectiveness of the intervention, with the potential for patients to have better outcomes with avoidance of surgery and long-term problems.
